# Prenatal Exposure to Cigarette Smoke and Anogenital Distance at 4 Years in the INMA-Asturias Cohort

**DOI:** 10.3390/ijerph18094774

**Published:** 2021-04-29

**Authors:** Miguel García-Villarino, Rocío Fernández-Iglesias, Isolina Riaño-Galán, Cristina Rodríguez-Dehli, Izaro Babarro, Ana Fernández-Somoano, Adonina Tardón

**Affiliations:** 1 Spanish Consortium for Research on Epidemiology and Public Health (CIBERESP), Monforte de Lemos Avenue 3-5, 28029 Madrid, Spain; miguelvillarino@outlook.es (M.G.-V.); rocio.fdez.iglesias@gmail.com (R.F.-I.); isolinariano@gmail.com (I.R.-G.); atardon@uniovi.es (A.T.); 2 Unit of Molecular Cancer Epidemiology, Department of Medicine, University Institute of Oncology of the Principality of Asturias (IUOPA)—University of Oviedo, Julián Clavería Street s/n., 33006 Oviedo, Spain; 3 Instituto de Investigación Sanitaria del Principado de Asturias (ISPA), Roma Avenue s/n., 33001 Oviedo, Spain; crdehli@yahoo.es; 4 Servicio de Pediatría, Endocrinología Pediátrica, HUCA, Roma Avenue s/n., 33001 Oviedo, Spain; 5 Servicio de Pediatría, Hospital San Agustín, Heros Street, 4, 33410 Avilés, Spain; 6 Faculty of Psychology, University of the Basque Country, 20018 Donostia/San Sebastian, Spain; izaro.babarro@ehu.eus; 7 Biodonostia Health Research Institute, Group of Environmental Epidemiology and Child Development, 20014 Donostia/San Sebastian, Spain

**Keywords:** anogenital distance, maternal cigarette smoking, tobacco smoking, endocrine-disrupting chemicals

## Abstract

Smoking by women is associated with adverse pregnancy outcomes such as spontaneous abortion, preterm delivery, low birth weight, infertility, and prolonged time to pregnancy. Anogenital distance (AGD) is a sensitive biomarker of prenatal androgen and antiandrogen exposure. We investigated the effect of smoking and passive smoke exposure during pregnancy on anogenital distance in offspring at 4 years in the INMA-Asturias cohort (Spain). Women were interviewed during pregnancy to collect information on tobacco consumption, and anogenital distance was measured in 381 children: Anoscrotal distance in boys and anofourchetal distance in girls. We also measured maternal urinary cotinine levels at 32 weeks of pregnancy. We constructed linear regression models to analyze the association between prenatal smoke exposure and anogenital distance and adjusted the models by relevant covariates. Reported prenatal smoke exposure was associated with statistically significant increased anogenital index (AGI), both at week 12 of pregnancy (β = 0.31, 95% confidence interval: 0.00, 0.63) and at week 32 of pregnancy (β = 0.31, 95% confidence interval: 0.00, 0.63) in male children, suggesting altered androgenic signaling.

## 1. Introduction

Tobacco use and passive smoking during pregnancy can exert multiple effects on offspring, which may persist into adulthood [1,2]. Passive exposure to cigarette smoke in childhood is a global public health problem associated with respiratory symptoms, increased risk of invasive meningococcal disease, and high blood pressure [3,4,5]. In Spain, 3.2% of children aged up to 4 years and 7.7% of children aged 5–14 years are exposed to cigarette smoke in enclosed spaces [6], and the effects of exposure have been well-documented [2,7]. Some constituents of tobacco or cigarette smoke have endocrine-disrupting properties [8]. Adverse pregnancy outcomes such as spontaneous abortion [9], preterm delivery [10], and low birth weight [11] can result from maternal smoking, as well as female infertility [12] and prolonged time to pregnancy [13,14]. Cigarette smoking rates among women and during pregnancy vary by region and similar differences apply to passive smoke exposures, sometimes termed environmental tobacco smoke [15,16,17,18].

Animal and epidemiological studies have shown that in utero exposure to cigarette compounds, including cotinine [19], may affect developmental reproductive biology. Among men, prenatal tobacco exposure is associated with increased hypospadias [20], reduced testis size and semen quality [21], reduced sperm count [22], altered reproductive hormone levels, precocious puberty, and final height and body mass index (BMI) [23]. These findings support the hypothesis of endocrinological disruption [24,25] following intrauterine exposure to substances that behave as antiandrogenic endocrine-disrupting chemicals. In women, intrauterine exposure to tobacco has been linked to premature menopause [26], reduced fertility, and early puberty and menarche [12,27].

Anogenital distance (AGD) has been validated in epidemiological studies as an anthropometric marker of intrauterine exposure to antiandrogens and androgens [28,29]. During the critical period of early fetal life termed “masculinization programming window” (8–14 weeks of gestation), AGD can represent fetal androgenic activity [30] and may be a predictor of lifelong reproductive health [31]. There is growing evidence that in men, exposure to androgens lengthens AGD and exposure to antiandrogens shortens AGD. In mammals, AGD is longer in males and reflects in utero masculinization. Endocrine-disrupting chemicals such as polybrominated diphenyl ethers, phthalates, polychlorinated biphenyls, and bisphenol A have been linked to shorter AGD in male children [28,32,33,34,35,36,37]. Furthermore, AGD may be a biomarker of hyperandrogenemia in women [38,39]. By contrast, an androgenic environment during intrauterine life may induce masculinization and a longer AGD [40]. Other studies have shown that female infants with congenital adrenal hyperplasia [41] and women with higher testosterone levels [42] or multifollicular ovaries [43] have a longer AGD. Shorter AGD in boys appears to be inversely related to testosterone levels, volume and quality of sperm, cryptorchidism, and hypospadias [44,45,46,47,48,49].

To date, few studies have been conducted on the influence of prenatal tobacco exposure on AGD [50,51]. To our knowledge, only one epidemiological study has linked maternal smoking to longer weight-adjusted AGD in female infants [52], and another study linked gestational tobacco use to longer AGD in male fetuses [51]. In the present study, we examined the effect of maternal smoking and passive exposure to cigarette smoke during pregnancy on AGD in children aged 4 years.

## 2. Materials and Methods

### 2.1. Study Design and Participants

**Figure 1:**
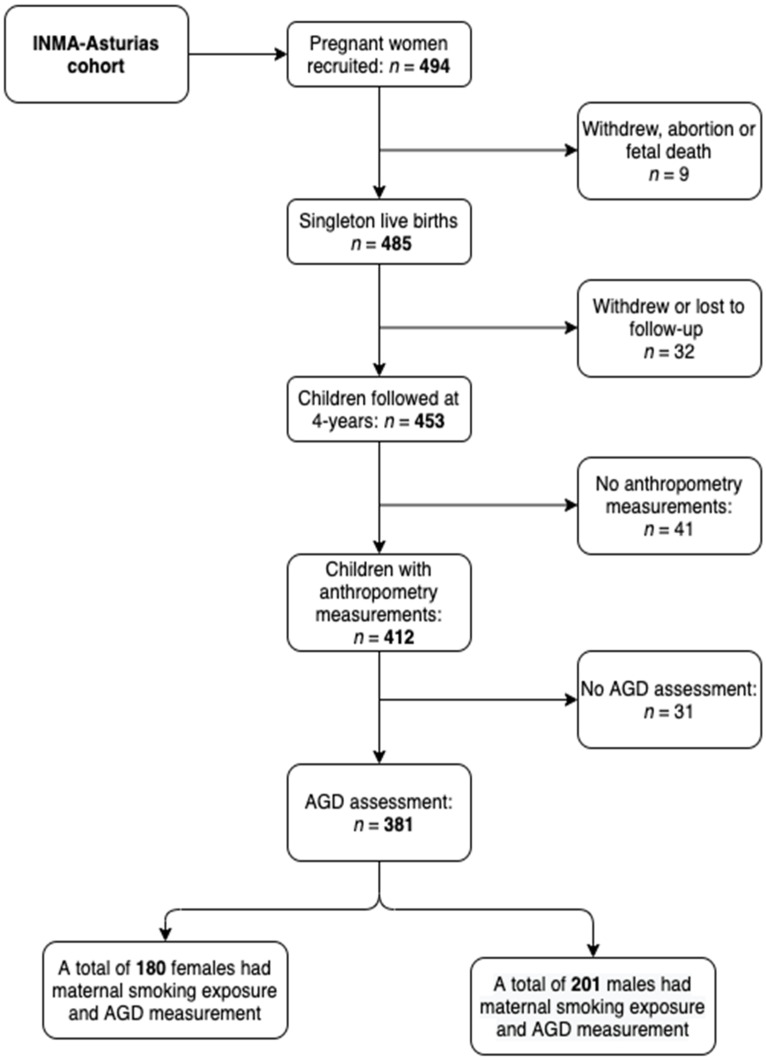
Flowchart of the study population in the present study from the INMA-Asturias cohort.

The study population was drawn from a cohort of mother-child pairs enrolled in the INMA (Infancia y Medio Ambiente (Environment and Childhood))-Asturias cohort and has been described in detail in our prior studies [53,54,55]. Four hundred and ninety-four pregnant women recruited between May 2004 and June 2007 agreed to participate. All participants met the inclusion criteria: aged ≥ 16 years, no assisted conception, enrollment at 10–13 weeks of gestation, singleton pregnancy, delivery scheduled at the reference hospital (San Agustin Hospital, Avilés, Spain), and no communication handicap. During the third trimester of pregnancy, 416 of the women completed a questionnaire on gestational tobacco use and other variables and provided urine samples for determination of urinary cotinine levels. A total of 485 children were born, and follow-up was carried out 4–5 years later on 453 children (93.4% participation rate) through questionnaires on diet, environmental health data, and sociodemographic variables (Figure 1). Two pediatricians examined 412 of those children and recorded anthropometric characteristics. The research protocol was approved by The Asturias Regional Clinic Research Ethics Committee; all women provided written informed consent prior to inclusion and then signed a second consent to enroll the children into the INMA-Asturias cohort.

### 2.2. Smoking Status

Questionnaires on tobacco use, including patterns of consumption, smoking history, and exposure to passive smoking were administered by one trained interviewer twice during pregnancy (around 12 and 32 gestational weeks). The following variables were extracted from the information collected in the questionnaires, we have obtained the following variables: Smoking at the beginning of pregnancy, smoking at week 12 of pregnancy, and smoking at week 32 of pregnancy (all yes/no). The number of daily cigarettes consumed during pregnancy was also collected. Maternal passive smoking exposure was assessed at week 32 and specified by location: home, work, or in leisure activities outside the home such as in bars, restaurants, and homes. The methodology regarding information on tobacco consumption has been reported previously [56,57,58].

### 2.3. AGD

AGD was measured according to methods described in detail elsewhere [59] and reported in our previous studies [34,35]. Vernier calipers were used to perform measurements in increments of 0.1 mm by two trained pediatricians. We measured AGD from the center of the anus to the posterior convergence of the fourchette in girls and from the center of the anus to the junction of the smooth perineal skin with the rugated skin of the scrotum in boys. The pediatricians faced the children and made independent measurements of AGD using one digital caliper. To assess inter-examiner variability, pediatricians took independent measurements of 10% of the entire sample using the same protocol. The outputs did not differ substantially, so only one measurement was noted.

### 2.4. Urinary Cotinine Levels

Urine samples were collected during the third trimester in 100-mL polyethylene containers and stored at −20 °C. One aliquot from each of the participants was analyzed by the Public Health Laboratory of Bilbao (Spain). A competitive enzyme immunoassay using commercial microplate test kits (OraSure Technologies, Inc., Bio-Rad Laboratories, Hercules, CA, USA) was performed to determine salivary cotinine adapted for urine samples using urine controls (0, 2.5, 10, and 50 ng/mL, Bio-Rad, Hercules, CA, USA). Samples with urinary cotinine levels above 50 ng/mL were diluted. The technique was validated using a certified reference material (EPA/NIST Reference Material 8444) to assess the repeatability and reproducibility. The quantification limit was 4.0 ng/mL, and the coefficients of repeatability and reproducibility were 7% and 10%, respectively [56].

### 2.5. Potential Confounders

The following maternal variables were considered potential confounders from the findings of prior studies: age, pre-pregnancy weight (self-reported), education (primary, secondary, or university), maternal social class (I–II (highest), III, or IV–V (lowest)), gestational age (weeks) at delivery, parity (1, 2, or ≥3), and pre-pregnancy BMI. Pre-pregnancy BMI was obtained by dividing self-reported pre-pregnancy weight by height at week 12 and was categorized as underweight (<18.5 kg/m^2^), normal (18.5–24.9 kg/m^2^), overweight (25.0–29.9 kg/m^2^), or obese (≥30 kg/m^2^). Child covariates considered potential confounders were height and BMI at 4 years.

### 2.6. Statistical Analysis

Demographic characteristics of the participants were expressed using counts and percentages for categorical variables and means and standard deviation (SD) for continuous variables. Following the procedure developed by Swan and colleagues, we calculated the anogenital index (AGI) as AGD divided by weight at age of examination [28]. Linear regression models were constructed to estimate and quantify the association between AGI at 4 years, which was treated as the dependent variable, and each one of the prenatal smoke exposure variables, which were treated as the independent variables. Bivariate analyses were conducted to identify the variables related to both AGI and prenatal smoke exposure, and those with a *p*-value < 0.2 were considered potential covariates. For this bivariate analysis, differences between continuous variables were evaluated using Spearman correlation coefficients, and differences between categorical variables were evaluated by *x*^2^ or Fisher’s exact test, as appropriate. Differences between continuous and categorical variables were analyzed by t-Student’s *t*-test or analysis of variance for normally distributed continuous variables and by the Mann–Whitney and Kruskal–Wallis tests for continuous variables that were not normally distributed. Models were first adjusted by child height at 4 years, maternal weight gain during pregnancy, and maternal pre-pregnancy BMI. Other potential covariates were selected from the literature using direct acyclic graphs and selecting the minimally sufficient adjustment set [60] (maternal age, gestational age at delivery, parity, maternal education level, and social class). When the associations between maternal smoking and AGI at 4 years was evaluated, the model was adjusted by the variables for passive smoking and vice versa. To avoid overfitting, only covariates that modified the exposure coefficient by 10% or more following forward stepwise regression were included in the final model.

Cotinine level (log-transformed) was included in the models as a continuous variable and as a categorical variable with 27 ng/mL as the cut-off point [56]. All statistical analyses were stratified by child sex. Linear regression models were validated by testing the normality, homoscedasticity, and independence of the residuals, and the variance inflation factor was used to study the multicollinearity of the regression models.

For sensitivity analyses, we evaluated the association between AGI at 4 years and each prenatal smoke exposure variable using the same adjusted model and including all statistically significant covariates for each child sex. We also analyzed the association between cotinine levels a categorical variable and AGI at 4 years using various cut-off points [61]. Finally, we evaluated the association between maternal consumption of cigarettes during pregnancy and AGI at 4 years to test a dose–response relationship. All analyses and graphics were performed in R 3.6.2 (R Development Core Team, Vienna, Austria) [62], and *p* < 0.05 was considered as statistically significant.

## 3. Results

Table 1 lists the characteristics of study participants. After excluding participants who withdrew, were lost to follow-up, underwent abortions, miscarried, or did not have complete exposure and outcome data, the analysis included 381 mother–child pairs (Figure 1). The socioeconomic and anthropometric measures of the children included in the analysis did not differ substantially from those of children who were excluded (*n* = 114, Table S1). For male children, the mean maternal age was 31.9 years (range: 19–42 years) and mean (SD) maternal height and weight were 162.4 (5.61) cm and 62.3 (11.31) kg, respectively. Approximately 30% of mothers were overweight or obese, and 42% had a university education. With regard to tobacco use, 28.5% were occasional or regular smokers at the beginning of the pregnancy, but only 17.7% reported smoking at week 12 of pregnancy and 17.2% at week 32. Among the women who reported smoking at the beginning of pregnancy, the average number of cigarettes consumed per day was 13. This consumption was reduced to eight cigarettes per day on week 12 of pregnancy and six on week 32. Half (51.5%) of the women were not exposed to passive smoking during pregnancy, 36% were exposed to one source, and 12.5% were exposed to more than one source. The mean maternal urinary cotinine level was 351.1 ng/mL. The use of a valid cut-off point is important in a reliable marker of tobacco smoking such as urinary cotinine that can discriminate nonsmokers from regular or occasional smokers during pregnancy are crucial issues. In 74.1% of women, urinary cotinine levels were under our cut-off point of 27 ng/mL (Figure S1). Descriptive data were similar among female children (Table 1). No disorders or genital malformations were detected in the 381 children. The mean AGD in boys was 33.92 (11.37) mm (range: 25–62 mm) and the mean (SD) AGI was 1.83 (0.63) mm/kg. In girls, the mean AGD was 17 mm (range: 13–90 mm) and mean AGI was 0.96 (0.27) mm/kg (Table 1).

**Figure 2:**
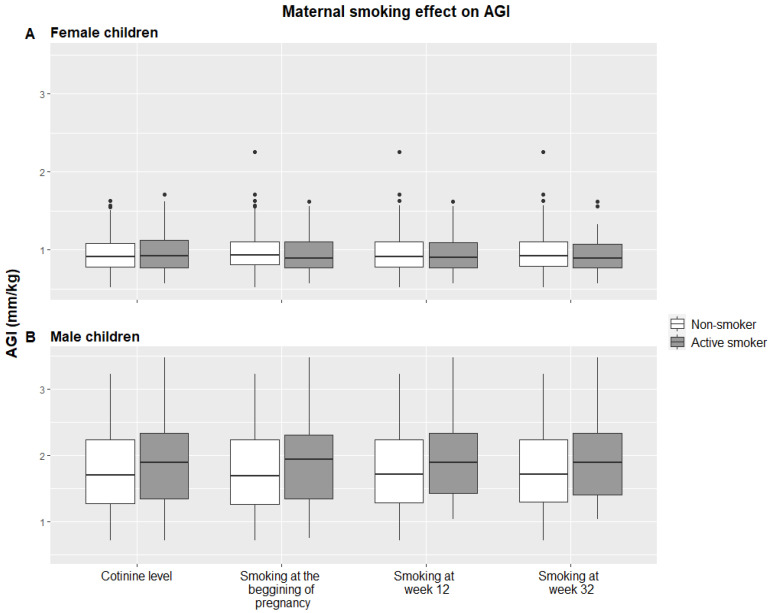
The effect of maternal smoking and anogenital distance in children from the INMA–Asturias cohort at 4 years.

Figure 2 shows the AGI stratified by sex and its relationship with the different variables of exposure to tobacco. In boys, but not girls, the AGI was slightly larger in those who had been exposed prenatally to tobacco. The difference in AGI means between children with mothers whose cotinine levels ≥ 27 ng/mL and < 27 ng/mL was 0.12 mm/kg (95% confidence interval (CI): −0.10; 0.34). The difference in AGI means between children with mothers who smoked at the beginning of pregnancy and those who did not was 0.13 mm/kg (95% CI: −0.06; 0.33). The difference in AGI means between children with mothers who smoked at week 12 of pregnancy and those who did not was 0.20 mm/kg (95% CI: −0.04; 0.45). The difference in AGI means between children with mothers who smoked at week 32 of pregnancy and those who did not was 0.19 mm/kg (95% CI: −0.06; 0.44). However, in girls, we did not observe any differences in AGI means between these groups (cotinine levels ≥ 27 ng/m vs. cotinine level < 27 ng/mL: 0.03 mm/kg (95% CI: −0.08; 0.14); mothers who smoke at the beginning of pregnancy vs. those who do not: 0.005 mm/kg (95% CI: −0.00; 0.09); mothers who smoke at week 12 vs. those who do not: <0.001 mm/kg (95% CI: −0.11; 0.10), and mothers who smoke at week 32 vs. those who do not: <0.001 mm/kg (95% CI: −0.12; 0.09)).

Table 2 summarizes the variables affecting AGI in children. Maternal reported smoking at week 12 and at week 32 of pregnancy was associated with an increase of 0.31 mm/kg (95% confidence interval (CI): 0.00, 0.63) in boys. We also observed a positive borderline statistically significant association between smoking at the beginning of pregnancy and the AGI (β = 0.20, 95% CI: −0.05, 0.46) in boys. In girls, we observed a marginal association between maternal urinary cotinine levels (continuous) and AGI (β = 0.01, 95% CI: 0.00, 0.03). We did not identify associations with passive smoking exposure and AGI for either sex.

## 4. Discussion

We studied the association between maternal smoking or exposure to passive smoking during pregnancy and AGI in children at 4 years and found that maternal smoking during the third trimester of pregnancy was associated with higher AGI in boys. To our knowledge, this is one of the first epidemiological studies in the literature that assessed gestational tobacco use and its effects on AGD.

Prenatal exposure to cigarette smoke is one modifiable cause of intrauterine growth restriction [63]. In the INMA cohort, associations between prenatal exposure to maternal smoking or maternal exposure to secondhand smoke and child BMI in the first 4 years of life have been observed [64]. In addition, fetal growth has been shown to be restricted by maternal smoking during pregnancy [65]. Tobacco use during pregnancy has been linked to a longer AGD and small size at birth in female newborns [52], although without correlating with the number of cigarettes smoked. However, another study reported that shorter AGD was associated with body weight and length at birth [50]. Adjusting for weight may restrict the use of AGD measurements, so AGD values should be normalized when assessing exposure to compounds with endocrine-disrupting properties such as cigarette smoke or persistent and non-persistent organic pollutants.

Fowler and colleagues reported that tobacco consumption during the second trimester of pregnancy was associated with longer AGD in male newborns [50] and after birth [51]. Gestational exposure to tobacco is known to negatively affect reproductive health in men (e.g., infertility, reduced testicular weight, and sperm count) [66,67]. Our findings are consistent with those of Fowler et al., indicating that in the male fetus, there is a dysregulation of masculinization just after the testosterone peak occurs [68]. The function of human fetal Leydig cells during the second trimester of pregnancy is probably dependent on stimulation via the chorionic gonadotropin/luteinizing hormone receptor; serum levels of human chorionic gonadotropin peak around weeks 10–14 of gestation and then decline [68,69]. A plausible explanation is that androgen-dependent development of the genital structure may be altered in children of smoking women [51], suggesting that exposure to cigarette smoke in utero may be associated with poor androgenic action in boys [52]. In the same way, the increase in AGD we found in smoke-exposed boys may have been triggered immediately after the peak time of androgen action.

Smoke-induced changes in AGD during the second trimester remotely seems to be caused by direct changes in androgen levels, and tobacco consumption does not appear to affect fetal testosterone levels [67]. Nonetheless, the circulating testosterone levels could not be the most effective indicator for measuring the androgen exposure to the external genitalia given that androgen synthesis has been shown as a relevant alternative [70]. In this regard, a more complete analysis of the circulating androgens and the effects of maternal smoking in male fetus would be necessary to fully characterize the impact of smoking on steroidogenesis. Otherwise, maternal smoking during pregnancy has an influence on the fetuses’ AGD directly over interaction with the effects of androgens or other endocrine systems. This can be through effects on androgen receptor expression or through activity in the region of the external genitalia [71]. Consequently, alterations in hCG levels appear to be associated with maternal smoking and be sex specific. Moreover, hCG has been linked to adverse consequences that might be generated by some EDCs such as phthalates [72]. On the other hand, gonadotropins appear to play an important role in effects on AGD that are not dependent on androgen as an intermediate. In addition, tobacco smoke contains compounds that can act as activating ligands for aryl hydrocarbon receptor (AHR), such as polycyclic-chromatic hydrocarbons, and studies in rodents have shown that these are among the active substances that affect female reproductive development [73]. In the same way, adverse effects in the human fetal ovary have been shown to be associated with changes in fetal AHR signaling [74]. The effects of AHR stimulation have not been established yet in tissues that respond to androgens, such as the external genitalia, and it could be possible that the AHR system is involved in some way in the association between tobacco use during pregnancy and altered AGI.

In pregnant women, tobacco use may lower estrogen levels and increase androgen levels [75]. Placental aromatase regulates the conversion of testosterone to estradiol and is inhibited by cigarette smoke, which therefore exerts an estrogenic effect [76,77]. In girls, only one study reported that maternal smoking during pregnancy was associated with longer weight-adjusted AGD [52]; these findings are in agreement with our analysis, as we found that urinary cotinine levels were positively associated with the AGI in our female dataset. This suggests that estrogens affect the development of the external genitalia in women. The biological mechanism may be increased estrogenic activity and not the androgenic effects seen in men. Prenatal exposure to some endocrine-disrupting chemicals has been shown to affect AGD through estrogenic pathways [38]. We previously reported that polychlorinated biphenyl 101 and dichlorodiphenyltrichloroethane and its derivates increased the AGI [35], and another group showed that maternal exposure to bisphenol A shortened the AGD in female newborns [37].

Several limitations should be noted. First, our information on tobacco use during pregnancy was self-reported and may not be reliable; although we validated the information against urinary cotinine levels and observed a positive borderline statistically significant association between cotinine levels and AGI in female children. By contrast, no such association was observed in male children, but the regression coefficient was negative, suggesting that a shorter AGI may be the result of exposure to other endocrine-disrupting chemicals [28,34,35,36] and in contrast to what we observed when analyzing self-reported smoking variables. Moreover, maternal smoking status was recorded during the third trimester of pregnancy, which may have led to some misclassification of the exposure early in pregnancy. Furthermore, it should be noted that we cannot evaluate the time window of pregnancy with greater sensitivity. This is due to the fact that the pregnant women’s exposure is essentially the same at weeks 12 and 32. Therefore, we can only conclude that the differences in AGI observed in male children are a consequence of the exposure during pregnancy. To sum up, the effects of the first and the third trimesters are indistinguishable. Second, two pediatricians performed the AGD measurements, leading to possible variability in the results. Unfortunately, only one AGD measurement per child was recorded. We tested inter-examiner reliability in 10% of samples and found no substantial variability. Third, we do not have data on testosterone levels in the children either prenatally or perinatally; to date, only a few studies have assessed the association between AGD and hormone levels at birth [78,79] or later in childhood [80]. One study reported that testosterone and estradiol measured in cord blood were not correlated with AGD [79], which is a finding corroborated by another study that suggested that AGD is unaffected by androgens [78]. By contrast, maternal smoking during pregnancy has been suggested to alter AGD through direct effects in androgen levels [50,51]. In addition, factors other than circulating estrogen or androgens may be responsible for alterations in the structure of external genitals [52], and autocrine/paracrine regulation may influence the levels of maternal or fetal hormones [81]. Therefore, we hypothesize that the threshold concentrations of some exogenous chemicals may account for the discrepancies in the literature. However, our study has notable strengths: We measured AGD at an age that has not previously been investigated, and our research used a well-regulated birth cohort. To date, studies on gestational tobacco exposure focused on measuring AGD at birth [50,51,52].

## 5. Conclusions

Our findings provide evidence that prenatal smoke exposure is associated with larger AGI in male children and are consistent with previous reports in the epidemiological literature. AGI was longer in smoke-exposed male children, suggesting altered androgenic signaling during the first and third trimester of pregnancy and during the gestational window of masculinization that affects genital development.

## Figures and Tables

**Table 1:** Characteristics of mothers and children enrolled in the INMA-Asturias cohort study.

Variables	Female Children (*n* = 180)			Male Children (*n* = 201)		
**Child characteristics**	***n***	***%***	**Mean (SD)**	***n***	***%***	**Mean (SD)**
AGD at 4 years (mm)	180		17.00 (4.89)	201		33.92 (11.37)
AGI at 4 years (mm/kg)	180		0.96 (0.27)	201		1.83 (0.63)
Birth weight (kg)	180		3.17 (0.48)	201		3.34 (0.45)
Birth length (cm)	180		49.23 (2.18)	201		49.99 (2.1)
Weight at 4 years (kg)	180		17.91 (2.78)	201		18.86 (2.98)
Height at 4 years (cm)	180		104.67 (4.53)	201		107.05 (4.45)
BMI at 4 years (kg/m^2^)	180		16.29 (1.79)	201		16.38 (1.79)
**Maternal characteristics**						
Age (years)	180		31.73 (4.19)	201		31.96 (4.40)
Gestational age (week)	180		39.57 (1.68)	201		39.35 (1.52)
Pre-pregnancy BMI	180			201		
Underweight (<18.5 kg/m^2^)	3	1.67		11	5.47	
Normal (18.5–24.9 kg/m^2^)	122	67.78		129	64.18	
Overweight (25.0–29.9 kg/m^2^)	43	23.89		42	20.90	
Obese (≥30 kg/m^2^)	12	6.67		19	9.45	
Weight (kg)	180		62.74 (11.11)	201		62.33 (11.31)
Height (cm)	180		162.62 (5.60)	201		162.48 (5.61)
Weight gain (kg)	176		13.36 (5.55)	195		13.99 (4.8)
Education	180			201		
Primary	32	17.78		32	15.92	
Secondary	83	46.11		84	41.79	
University	65	36.11		85	42.29	
Social class	180			200		
I–II (highest)	35	19.44		54	27.00	
III	37	20.56		44	22.00	
IV–V (lowest)	108	60.00		102	51.00	
Parity	180			201		
One	106	58.89		126	62.69	
Two	65	36.11		68	33.83	
Three or more	9	5.00		7	3.48	
Cotinine (ng/mL)	167		301.16 (806.92)	178		351.1 (838.64)
Cotinine	167			178		
<27 ng/ml	129	77.25		132	74.16	
≥27 ng/ml	38	22.75		46	25.84	
Cigarettes/day at the beginning of pregnancy ^a^	172		11.92 (9.01)	192		13.19 (10.04)
Smoking at the beginning of pregnancy	172			192		
No	126	73.26		138	71.88	
Yes	46	26.74		54	28.12	
Cigarettes/day at week 12 of pregnancy ^a^	169		6.74 (4.93)	192		7.74 (6.62)
Smoking at week 12 of pregnancy	171			192		
No	145	84.80		158	82.29	
Yes	26	15.20		34	17.71	
Cigarettes/day at week 32 of pregnancy ^a^	172		6.83 (5.12)	192		5.77 (3.61)
Smoking at week 32 of pregnancy	172			192		
No	147	85.47		159	82.81	
Yes	25	14.53		33	17.19	
Passive smoke exposure during pregnancy	172			192		
No exposure	96	55.81		99	51.56	
One between home/work/rest/leisure	53	30.81		69	35.94	
More than one between home/work/rest/leisure	23	13.37		24	12.50	

^a^ Mean and standard deviation were calculated only for those women that reported smoke in this period; BMI, body mass index.

**Table 2:** Association between prenatal exposure to tobacco and anogenital index in 4-year-old children.

	Female Children (*n* = 180)			Male Children (*n* = 201)		
	*β*	95% CI	*p*-Value	*β*	95% CI	*p*-Value
**Active smoke exposure**						
Cotinine (continuous) ^1^	0.01	(0.00, 0.03)	0.07	−0.01	(−0.05, 0.03)	0.66
Cotinine (categorical) ^2^	0.06	(−0.05, 0.17)	0.30	0.11	(−0.18, 0.36)	0.45
Cigarettes/day at the beginning of pregnancy ^2^	0.00	(0.00, 0.01)	0.54	0.01	(−0.00, 0.03)	0.17
Smoking at the beginning of pregnancy ^3^	0.00	(−0.10, 0.10)	1.00	0.20	(−0.05, 0.46)	0.12
Cigarettes/day at week 12 of pregnancy ^2^	0.00	(−0.02, 0.02)	0.90	0.02	(−0.01, 0.04)	0.27
Smoking at week 12 of pregnancy ^3^	0.01	(−0.12, 0.13)	0.87	0.31	(0.00, 0.63)	0.05
Cigarettes/day at week 32 of pregnancy ^2^	0.00	(−0.02, 0.01)	0.85	0.02	(−0.03,0.06)	0.46
Smoking at week 32 of pregnancy ^3^	0.00	(−0.13, 0.13)	0.99	0.31	(0.00, 0.63)	0.05
**Passive smoke exposure ^4^**						
One between home/work/rest/leisure	0.04	(−0.06, 0.14)	0.45	−0.13	(−0.33, 0.08)	0.23
More than one between home/work/rest/leisure	0.02	(−0.11,0.15)	0.81	−0.02	(−0.35, 0.32)	0.93

***β***: Regression coefficient; CI: Confidence interval. ^1^ Cotinine was log-transformed. Adjusted by height at four years, maternal weight gain during pregnancy, pre-pregnancy BMI, mother passive smoke exposure during pregnancy. ^2^ Adjusted by height at 4 years, maternal weight gain during pregnancy, pre-pregnancy BMI, gestational age (week), maternal age, social class, parity, mother passive smoke exposure during pregnancy. ^3^ Adjusted by height at 4 years, maternal weight gain during pregnancy, pre-pregnancy BMI, gestational age (week), maternal age, maternal education, social class, parity, mother passive smoke exposure during pregnancy. ^4^ Reference category: no exposure. Adjusted by height at 4 years, maternal weight gain during pregnancy, pre-pregnancy BMI, gestational age (week), maternal age, maternal education, social class, parity, and smoking at week 12.

## Data Availability

Not applicable.

## Supplementary Materials

The following are available online at https://www.mdpi.com/article/10.3390/ijerph18094774/s1, Table S1: Selected characteristics of the study population. Figure S1: Directed acyclic graph showing the minimal sufficient adjustment set. Figure S2: Regression coefficients and 95% confidence intervals for cotinine cut-off points.
