# Supplementary material for: Prenatal Exposure to Cigarette Smoke and Anogenital Distance at 4 Years in the INMA-Asturias Cohort
Source: Int J Environ Res Public Health. 2021 Apr 29;18(9):4774. doi: 10.3390/ijerph18094774 (PMC8124891; doi:10.3390/ijerph18094774)
Supplement: Supplementary file 1 [file ijerph-18-04774-s001.zip › ijerph-1200637-supplementary.pdf]

Table S1. Selected characteristics of the study population.

Variables	Sub-Sample with Available Data on AGD (n = 381)			Excluded (n = 114)			p-Value
**Child characteristics**	n	%	Mean (SD)	n	%	Mean (SD)	
Gender	381			31			0.97
Female	180	47.24		14	45.16		
Male	201	52.76		17	54.84		
Birth weight (kg)	381		3.26 (0.47)	102		3.27 (0.53)	0.46
Birth length (cm)	379		49.63 (2.17)	101		49.77 (1.93)	0.55
Weight at 4 years (kg)	381		18.41 (2.92)	28		18.41 (2.22)	0.67
Height at 4 years (cm)	381		105.93 (4.64)	28		106.17 (4.64)	0.79
BMI at 4 years (kg/m^2)	381		16.34 (1.79)	28		16.33 (1.61)	0.87
**Maternal characteristics**							
Age (years)	381		31.85 (4.3)	104		30.29 (4.47)	0.01
Gestational age (week)	381		39.45 (1.6)	104		39.33 (2.26)	0.75
Pre-pregnancy BMI	381			104			1
Underweight (<18.5 kg/m2)	14	3.67		3	2.88		
Normal (18.5–24.9 kg/m2)	251	65.88		69	66.35		
Overweight (25.0–29.9 kg/m2)	85	22.31		23	22.12		
Obese (≥30 kg/m^2)	31	8.14		9	8.65		
Weight (kg)	381		62.52 (11.2)	104		63.41 (12.54)	0.57
Height (cm)	368		162.19 (5.81)	33		162.68 (6.87)	0.28
Weight gain (kg)	367		13.69 (5.17)	33		14.25 (5.64)	0.37
Education	381			104			0.14
Primary	64	16.8		24	23.08		
Secondary	167	43.83		49	47.12		
University	150	39.37		31	29.81		
Social class	380			104			0.58
I–II (highest)	89	23.42		20	19.23		
III	81	21.32		21	20.19		
IV–V (lowest)	210	55.26		63	60.58		
Parity	381			104			0.3
One	232	60.89		64	61.54		
Two	133	34.91		32	30.77		
Three or more	16	4.2		8	7.69		
Cotinine (ng/mL)	345		326.92 (822.62)	83		549.65 (1235.41)	0.06
Cotinine	345			83			0.24
< 27 ng/ml	261	75.65		57	68.67		
≥ 27 ng/ml	84	24.35		26	31.33		
Cigarettes/day at the beginning of pregnancy	364		12.61 (9.56)	92		11.82 (7.89)	0.86
Smoking at the beginning of pregnancy	364			92			0.52
No	264	72.53		63	68.48		
Yes	100	27.47		29	31.52		
Cigarettes/day at week 12 of pregnancy	361		7.33 (5.95)	92		7.19 (5.45)	0.84
Smoking at week 12 of pregnancy	363			92			0.08
No	303	83.47		69	75		
Yes	60	16.53		23	25		
Cigarettes/day at week 32 of pregnancy	364		6.23 (4.31)	92		7.24 (5.21)	0.52
Smoking at week 32 of pregnancy	364			92			0.16
No	306	84.07		71	77.17		

Yes	58	15.93	21	22.83	
Passive smoke exposure during pregnancy	364		92		0.11
No exposure	195	53.57	38	41.3	
One between home/work/rest/leisure	122	33.52	38	41.3	
More than one between home/work/rest/leisure	47	12.91	16	17.39	

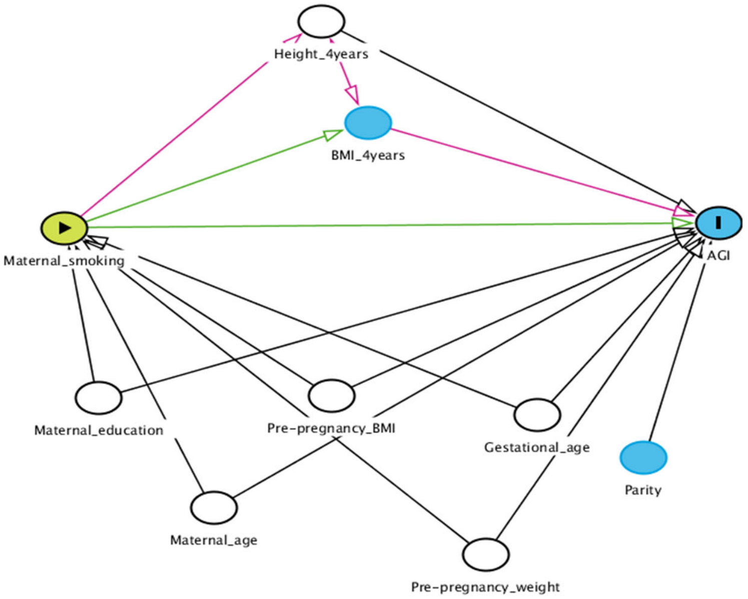

Figure S1. Directed acyclic graph showing the minimal sufficient adjustment set.

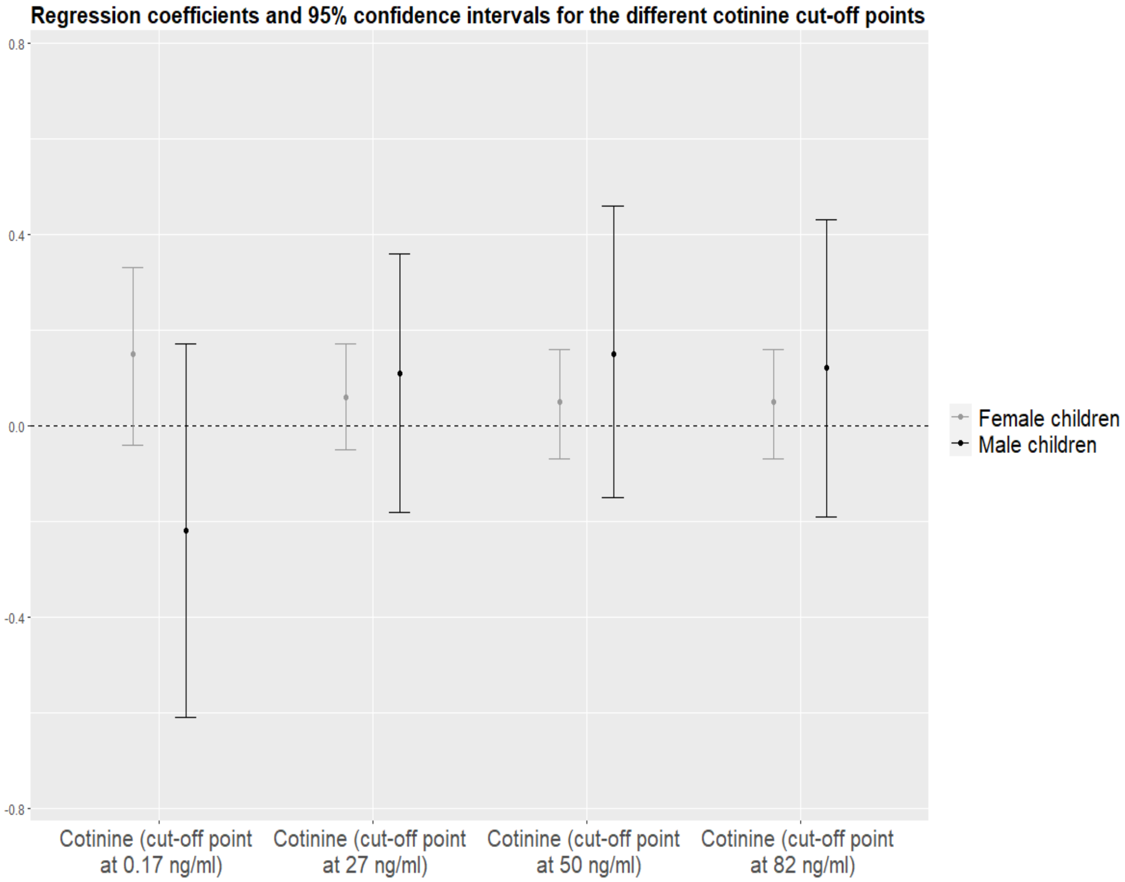

Figure S2. Regression coefficients and 95% confidence intervals for cotinine cut-off points. The reference category is the one with cotinine values lower than the indicated cut-off point in each case.
